# Pirogoff’s Operation

**Published:** 1861-12

**Authors:** 


### PIROGOFF'S OPERATION.

As this operation at the ankle-joint has been recommended in an order from the Medical Director of the army, in preference to Chopart's or to amputation above the ankle, it may be interesting to our readers to see a description of that operation, as given by the author himself. He says:

"I commence my incision close in front of the outer malleolus, carry it vertically downwards to the sole of the foot, then transversely across the sole, and lastly obliquely upwards to the inner malleolus, where I terminate it a couple of lines anterior to the malleolus. Thus all the soft parts are divided at once quite down to the os calcis. I now connect the outer and inner extremity of this first incision by a second semilunar incision, the convexity of which looks forward, carried a few lines anterior to the tibio-tarsal articulation. I cut through all the soft parts at once down to the bones, and then proceed to open the joint from the front, cutting through the lateral ligaments, and thus exarticulate the head of the astragalus. I now place a small, narrow amputation saw obliquely upon the os calcis, behind the astragalus, exactly upon the sustentaculum tali, and saw through the os calcis, so that the saw passes into the first incision through the soft parts. Saw carefully, or the anterior surface of the tendo achillis, which is only covered by a layer of fat, and a thin fibrous sheath, might be injured. I separate the short anterior flap from the two malleoli, and saw through them at the same time close to their base. I turn this flap forwards, and bring the cut surface of the os calcis in opposition with the articular surface of the tibia. If the latter be diseased, it is sometimes necessary also to saw off from it a thin slice with the malleoli."

He thus sums up its absolute and relative merits:

"1. The tendo achillis is not dividid, and so we avoid all the disadvantages connected with its injury. 2. It also follows that the base of the posterior flap is not thinner than its apex, while the skin on the base of the flap remains ununited with the fibrous sheath of the tendo achillis. 3. The posterior flap is cap-like, as in Syme's method, and its form is therefore less favorable to a collection of pus. 4. The leg, after my operation, appears an inch and a half (sometimes even more) longer than in the three other operations (Syme, Baudens, Roux), because the remnant of the os calcis left in the flap, as it unites with the inferior extremities of the tibia and fibula, lengthens them by an inch and a half; and 5. Serves the patient as the point of support."—*Reporter*.

